# Supplementary material for: Disynaptic cerebrocerebellar pathways originating from multiple functionally distinct cortical areas
Source: eLife. 2020 Aug 14;9:e59148. doi: 10.7554/eLife.59148 (PMC7428308; doi:10.7554/eLife.59148)
Supplement: Supplementary file 1. — (A) Table listing the location and size of mono-trans-synaptic adeno-associated virus (AAV1.cre) injection sites per animal. (B) Table showing the quantification of resulting anterograde labeling in precerebellar nuclei following injection of AAV1.cre into target cortical regions. (C) Table showing the density of mossy fibers within each cerebellar region following injection of AAV1.cre into target cortical regions. (D) Table showing the quantification of resulting retrograde labeling following injection of Cholera-toxin B retrograde tracer into target cerebellar regions. (E) Exact p-values associated with Figure 5, Figure 6, and Figure 7—figure supplement 3. [file elife-59148-supp1.docx]

**Supplementary file 1**

| **Cortical**  **Region** | **Injection**  **depth**  **[µm]^1^** | **Injection**  **width**  **[µm]^2^** | **Injection**  **length (r-c)**  **[µm]^3^** | **Injection**  **volume**  **[mm^3^]** |
| --- | --- | --- | --- | --- |
| M1 (♂) | 650 | 1100 | 360 | 0.20 |
| M1 (♂) | 600 | 540 | 360 | 0.09 |
| M1 (♀) | 550 | 1100 | 280 | 0.15 |
| S1 (♂) | 580 | 900 | 320 | 0.07 |
| S1 (♂) | 630 | 720 | 360 | 0.13 |
| S1 (♀) | 520 | 950 | 400 | 0.16 |
| PPC (♂) | 480 | 650 | 360 | 0.09 |
| PPC (♂) | 790 | 640 | 280 | 0.11 |
| PPC (♀) | 490 | 670 | 280 | 0.07 |
| V1 (♀) | 640 | 650 | 280 | 0.12 |
| V1 (♂) | 450 | 560 | 320 | 0.06 |
| V1 (♂) | 600 | 640 | 320 | 0.10 |
| A1 (♀) | 710 | 560 | 360 | 0.11 |
| A1 (♀) | 550 | 700 | 320 | 0.11 |
| A1 (♂) | 450 | 850 | 320 | 0.10 |
| AuD (♂) | 600 | 800 | 320 | 0.12 |
| AuD (♂) | 800 | 760 | 360 | 0.17 |
| AuD (♀) | 650 | 550 | 280 | 0.08 |

**Supplementary file 1A. Location and size of mono-trans-synaptic adeno-associated virus (AAV1.cre) injection sites.** Table lists the cortical region where AAV1.cre was injected in each animal (3 mice per area; gender in brackets): primary motor (M1), primary somatosensory (S1), posterior parietal association (PPC), primary visual (V1), primary auditory (A1), or dorsal auditory (AuD) cortex, diameter of AAV injection sites (^1^measured perpendicular to cortical layers, ^2^measured parallel to cortical layers, ^3^measured across coronal sections) as well as their volume.

| **Injection site (gender)** | **Pn** | **Extra-pontine precerebellar nuclei** | | | | | | | | | | | **Total** | **Mossy fibers** |
| --- | --- | --- | --- | --- | --- | --- | --- | --- | --- | --- | --- | --- | --- | --- |
|  |  |  | | **Vestibular nuclei** | | |  | | | | **Extra- pontine** Total | **Extra- pontine**  % of All | **All** |  |
|  |  | **Ru** | **RtTg** | **M_PC_ M_MC_ Su** | | | **CN** | **Sp5l** | **Mx** | **LRt** |  |  |  |  |
| M1 (♂) | 94 | 2 | 2 | 1 | 2 |  |  | 44 | 1 |  | 52 | 36 % | 146 | 2406 |
| M1 (♂) | 206 | 14 |  | 3 | 5 | 4 |  | 104 |  |  | 130 | 39 % | 336 | 25616 |
| M1 (♀) | 80 | 2 | 2 | 3 | 5 | 2 |  | 51 | 6 |  | 71 | 47 % | 151 | 9024 |
| **Avg** |  |  |  |  |  |  |  |  |  |  |  | **40 ± 3 %** |  |  |
| S1 (♂) | 18 | 4 | 39 | 10 |  | 5 |  | 21 | 18 | 5 | 102 | 85 % | 120 | 17634 |
| S1 (♂) | 83 | 12 | 30 | 10 |  | 1 |  | 49 | 4 | 9 | 115 | 58 % | 198 | 14573 |
| S1 (♀) | 46 | 2 | 23 | 3 |  |  |  | 58 | 3 | 4 | 93 | 67 % | 139 | 2613 |
| **Avg** |  |  |  |  |  |  |  |  |  |  |  | **70 ± 8 %** |  |  |
| PPC (♂) | 37 | 2 | 7 |  |  |  |  |  |  |  | 9 | 20 % | 46 | 587 |
| PPC (♂) | 92 |  | 2 |  |  |  |  |  |  |  | 2 | 2 % | 94 | 928 |
| PPC (♀) | 14 |  | 11 |  |  |  |  |  |  |  | 11 | 44 % | 25 | 678 |
| **Avg** |  |  |  |  |  |  |  |  |  |  |  | **22 ± 12 %** |  |  |
| V1 (♀) | 66 |  | 3 | 5 |  |  |  |  |  |  | 8 | 11 % | 74 | 1541 |
| V1 (♂) | 95 |  | 13 | 10 |  | 2 |  |  |  |  | 25 | 21 % | 120 | 1231 |
| V1 (♂) | 39 |  | 1 | 10 |  |  |  |  |  |  | 11 | 22 % | 50 | 465 |
| **Avg** |  |  |  |  |  |  |  |  |  |  |  | **18 ± 4 %** |  |  |
| A1 (♀) | 0 |  | 2 | 3 |  | 4 | 1 |  |  |  | 10 | 100 % | 10 | 1589 |
| A1 (♀) | 0 |  |  | 3 |  | 6 | 1 |  |  |  | 10 | 100 % | 10 | 788 |
| A1 (♂) | 0 |  |  | 11 |  | 4 | 2 |  |  |  | 17 | 100 % | 17 | 595 |
| **Avg** |  |  |  |  |  |  |  |  |  |  |  | **100 %** |  |  |
| AuD (♂) | 7 |  |  | 15 |  | 4 | 1 |  |  |  | 20 | 74 % | 27 | 690 |
| AuD (♂) | 13 |  |  | 3 |  |  |  |  |  |  | 3 | 19 % | 16 | 518 |
| AuD (♀) | 9 |  |  | 2 |  | 1 | 3 |  |  |  | 6 | 40 % | 15 | 538 |
| **Avg** |  |  |  |  |  |  |  |  |  |  |  | **44 ± 16 %** |  |  |
| **Total All** | **899** | **38** | **135** | **92** | **12** | **33** | **8** | **327** | **32** | **18** | **695** | **49 ± 8 %** | **1594** | **82014** |

**Supplementary file 1B: Quantification of resulting anterograde labeling in precerebellar nuclei following injection of mono-trans-synaptic adeno-associated virus (AAV1.cre) into target cortical regions.** Table lists the cortical region where AAV1.cre was injected in each animal (3 mice per area; gender in brackets): primary motor (M1), primary somatosensory (S1), posterior parietal association (PPC), primary visual (V1), primary auditory (A1), or dorsal auditory (AuD) cortex, followed by quantification of labeled cells observed within the pontine nuclei (Pn) and the extra-pontine precerebellar nuclei (red nucleus [Ru], reticulotegmental nucleus [RtTg], parvocellular [M_PC_] and magnocellular [M_MC_] part of the medial vestibular nucleus, superior vestibular nucleus [Su], cochlear nuclear complex [CN], interpolar part of the spinal trigeminal nucleus [Sp5I], matrix region x [Mx], lateral reticular nucelus [LRt]), the total cell count for all extra-pontine nuclei, the percentage of labeling in the extra-pontine nuclei out of all observed precerebellar labeling (including average for each target region, mean ± s.e.m.), and the total cell count for all precerebellar nuclei (pontine + all extra-pontine labeling). Finally, the total number of mossy fiber terminals observed in the cerebellum is listed for each injection site.

| **Cerebellar region** | **Cerebral cortex target injection site** | | | | | |
| --- | --- | --- | --- | --- | --- | --- |
|  | **M1** | **S1** | **PPC** | **V1** | **A1** | **AuD** |
| Ipsilateral |  |  |  |  |  |  |
| I | 0 | 5 ± 4 | 0 | 0 | 33 ± 26 | 0 |
| II | 0 | 22 ± 16 | 5 ± 2 | 4 ± 3 | 9 ± 7 | 3 ± 1 |
| III | 26 ± 10 | 44 ± 26 | 5 ± 2 | 10 ± 4 | 26 ± 12 | 3 ± 1 |
| IV/V | 45 ± 15 | 96 ± 41 | 10 ± 4 | 4 ± 2 | 20 ± 5 | 7 ± 2 |
| VI | 241 ± 133 | 75 ± 25 | 68 ± 36 | 14 ± 3 | 14 ± 3 | 15 ± 2 |
| VII | 34 ± 18 | 166 ± 105 | 2 ± 2 | 1 ± 1 | 1 ± 1 | 7 ± 6 |
| VIII | 1 ± 1 | 14 ± 12 | 2 ± 1 | 0 | 4 ± 2 | 11 ± 5 |
| IX | 5 ± 3 | 2 ± 1 | 2 ± 1 | 1 ± 1 | 6 ± 2 | 5 ± 2 |
| X | 56 ± 29 | 10 ± 7 | 10 ± 4 | 0 | 8 ± 4 | 8 ± 3 |
| Sim | 312 ± 148 | 47 ± 19 | 3 ± 2 | 7 ± 1 | 12 ± 2 | 6 ± 2 |
| Crus I | 323 ± 174 | 106 ± 21 | 16 ± 3 | 28 ± 4 | 31 ± 5 | 29 ± 4 |
| Crus II | 404 ± 212 | 37 ± 7 | 14 ± 5 | 7 ± 3 | 14 ± 2 | 14 ± 2 |
| PM | 254 ± 72 | 126 ± 61 | 12 ± 2 | 3 ± 1 | 10 ± 3 | 11 ± 3 |
| Cop | 16 ± 1 | 102 ± 41 | 6 ± 2 | 5 ± 0.4 | 4 ± 2 | 7 ± 3 |
| PFl | 50 ± 20 | 117 ± 16 | 29 ± 4 | 35 ± 7 | 20 ± 3 | 20 ± 6 |
| Fl | 0 | 25 ± 13 | 4 ± 3 | 8 ± 3 | 2 ± 1 | 0 |
| Contralateral |  |  |  |  |  |  |
| I | 19 ± 16 | 8 ± 7 | 0 | 0 | 0 | 9 ± 8 |
| II | 0 | 68 ± 40 | 1 ± 0.4 | 2 ± 1 | 3 ± 2 | 1 ± 1 |
| III | 31 ± 13 | 242 ± 92 | 6 ± 2 | 28 ± 15 | 144 ± 103 | 6 ± 0 |
| IV/V | 101 ± 34 | 864 ± 333 | 13 ± 3 | 10 ± 3 | 45 ± 19 | 15 ± 1 |
| VI | 181 ± 90 | 336 ± 115 | 28 ± 3 | 53 ± 24 | 71 ± 32 | 34 ± 8 |
| VII | 40 ± 18 | 232 ± 174 | 4 ± 3 | 2 ± 2 | 2 ± 2 | 8 ± 6 |
| VIII | 0 | 21 ± 18 | 0.4 ± 0.4 | 0.4 ± 0.4 | 5 ± 2 | 8 ± 5 |
| IX | 2 ± 1 | 8 ± 4 | 4 ± 3 | 1 ± 1 | 12 ± 3 | 7 ± 3 |
| X | 2 ± 1 | 3 ± 1 | 4 ± 2 | 5 ± 2 | 46 ± 15 | 16 ± 4 |
| Sim | 788 ± 367 | 197 ±4 | 4 ± 1 | 7 ± 2 | 10 ± 2 | 8 ± 2 |
| Crus I | 752 ± 359 | 572 ± 17 | 75 ± 32 | 99 ± 39 | 35 ± 5 | 41 ± 8 |
| Crus II | 1548 ± 930 | 124 ± 25 | 9 ± 1 | 10 ± 4 | 32 ± 5 | 22 ± 3 |
| PM | 690 ± 189 | 860 ± 382 | 8 ± 2 | 8 ± 2 | 31 ± 9 | 14 ± 2 |
| Cop | 37 ± 4 | 2211 ± 1008 | 9 ± 16 | 10 ± 6 | 17 ± 6 | 10 ± 5 |
| PFl | 303 ± 91 | 758 ± 213 | 90 ± 33 | 285 ± 87 | 33 ± 8 | 27 ± 2 |
| Fl | 8 ± 7 | 51 ± 32 | 14 ± 7 | 13 ± 6 | 11 ± 2 | 0.2 ± 0.04 |

**Supplementary file 1C. Density of mossy fibers within each cerebellar region following injection of mono-trans-synaptic adeno-associated virus (AAV1.cre) into target cortical regions.** Table lists the density of mossy fibers [mossy fibers/mm^3^] ± s.e.m. for each cerebellar region of the ipsilateral and contralateral hemisphere separately, after corresponding cortical injections in: primary motor (M1), primary somatosensory (S1), posterior parietal association (PPC), primary visual (V1), primary auditory (A1), or dorsal auditory (AuD) cortex. For each target cortical area, n = 3 mice.

| **Injection site (gender)** | **Pn** | **RtTg** | **VN** | **Sp5l** | **Mx** | **LRt** | **Extra-pontine**  Total |
| --- | --- | --- | --- | --- | --- | --- | --- |
| Lobule IV/V (♀) | 45 | 58 |  | 44 | 9 | 49 | 160 |
| Lobule IV/V (♂) | 521 | 245 |  |  | 12 | 77 | 334 |
| Lobule IV/V (♀) | 259 | 22 |  | 2 | 15 | 115 | 154 |
| **Total** | **825** | **325** |  | **46** | **36** | **241** | **648** |
| Lobule VI (♀) | 33 | 66 | 4 |  | 5 | 10 | 85 |
| Lobule VI (♂) | 615 | 270 | 19 |  |  |  | 289 |
| Lobule VI (♂) | 363 | 285 |  |  |  |  | 285 |
| **Total** | **1011** | **621** | **23** |  | **5** | **10** | **659** |
| Sim (♀) | 939 | 44 | 14 | 31 |  | 81 | 170 |
| Sim (♂) | 646 | 46 |  | 8 | 9 | 24 | 87 |
| Sim (♀) | 393 | 91 | 3 | 11 | 11 | 20 | 136 |
| **Total** | **1978** | **181** | **17** | **50** | **20** | **125** | **393** |
| Crus I (♀) | 711 | 79 |  | 100 |  | 28 | 207 |
| Crus I (♀) | 924 | 132 |  |  | 8 | 13 | 153 |
| Crus I (♂) | 980 | 222 |  | 58 | 1 | 37 | 318 |
| **Total** | **2615** | **433** |  | **158** | **9** | **78** | **678** |

**Supplementary file 1D: Quantification of resulting labeling following injection of Cholera-toxin B retrograde tracer into target cerebellar regions.** Table lists the cerebellar region where CTB was injected in each animal (3 mice per area; gender in brackets), followed by the quantification of labeled cells observed within the pontine nuclei (Pn) and extra-pontine precerebellar nuclei: reticulotegmental nucleus [RtTg], vestibular nucleus (VN), interpolar part of the spinal trigeminal nucleus [Sp5I], matrix region x [Mx], lateral reticular nucleus [LRt]). Finally, the total cell count for all extra-pontine nuclei is listed.

| Figure | Condition | p-value |
| --- | --- | --- |
| 5B | Crus I: M1-M1 vs. M1-Other | 0.952 |
|  | Crus I: S1-S1 vs S1-Other | 0.334 |
|  | Crus II: M1-M1 vs. M1-Other | 0.007 ** |
|  | Crus II: S1-S1 vs S1-Other | <0.001 *** |
|  | Sim: M1-M1 vs. M1-Other | 0.002 ** |
|  | Sim: S1-S1 vs S1-Other | <0.001 *** |
|  | PM & Cop: M1-M1 vs. M1-Other | 0.971 |
|  | PM & Cop: S1-S1 vs S1-Other | 0.002 ** |
| 6B | PFl: M1-M1 vs. M1-Other | 0.373 |
|  | PFl: S1-S1 vs S1-Other | 0.011 * |
|  | IV/V: M1-M1 vs. M1-Other | 0.376 |
|  | IV/V: S1-S1 vs S1-Other | 0.055 |
|  | VI: M1-M1 vs. M1-Other | 0.345 |
|  | VI: S1-S1 vs S1-Other | 0.011 * |
| 7 – suppl 2A | Pn: M1-M1 vs. M1-IV/V | 0.842 |
|  | Pn: S1-S1 vs. S1-Crus I | 0.661 |
|  | RtTg: S1-S1 vs. S1-Crus I | 0.998 |
|  | Sp5l: S1-S1 vs. S1-Crus I | 0.942 |
|  | LRt: S1-S1 vs. S1-Crus I | 0.137 |
|  | Mx: S1-S1 vs. S1-Crus I | 0.195 |
| 7 – suppl 2B | Pn: M1-M1 vs. M1-IV/V | <0.001 *** |
|  | Pn: S1-S1 vs. S1-Crus I | <0.001 *** |
|  | RtTg: S1-S1 vs. S1-Crus I | <0.001 *** |
|  | Sp5l: S1-S1 vs. S1-Crus I | 0.376 |
|  | LRt: S1-S1 vs. S1-Crus I | 0.009 ** |
|  | Mx: S1-S1 vs. S1-Crus I | 0.029 * |

**Supplementary file 1E. Exact p-values associated with Figure 5, Figure 6, and Figure 7 – figure supplement 3.** Table lists exact p-values for the figures indicated (*p<0.05, **p<0.01, ***p<0.001). Primary motor cortex (M1), primary somatosensory cortex (S1), cortical injections sites extraneous to M1 or S1 (Other), pontine nuclei (Pn), reticulotegmental nucleus (RtTg), interpolar part of the spinal trigeminal nucleus (Sp5I), matrix region x (Mx), lateral reticular nucleus (LRt).
